# Young Female With Gingival Lesion of Intraoral Osseous Choristoma: A Rare Case Report

**DOI:** 10.1155/crid/7706892

**Published:** 2025-05-21

**Authors:** Ahmad Khaled Alhakim, Shady Ahmed Moussa, Esam Halboub

**Affiliations:** ^1^Oral and Dental Surgery Department, PHCC, Doha, Qatar; ^2^Pediatric Dentistry Department, Zagazig University, Zagazig, Egypt; ^3^Maxillofacial Surgery and Diagnostic Sciences Department, College of Dentistry, Jazan University, Jazan, Saudi Arabia

**Keywords:** choristoma, gingiva, hamartoma, intraoral, osseous

## Abstract

Choristoma is a mass of tissue with normal histology similar to a part of the body that is different from the one in which it is located. It differs from the hamartomatous group of pathology which is normal tissue, but disorganized, found in an abnormal location. Documentation of the occurrence of osseous choristoma lesions in the oral cavity has been scarce, and most of the cases have been reported in the tongue. It is very seldom to arise from the interdental gingiva. Indeed, choristoma is considered a developmental anomaly, involving younger individuals, although many cases among older adults have been reported. The differential diagnoses include cartilaginous metaplasia, pleomorphic adenoma, salivary gland tissue, and the lesions of inflammatory or traumatic origin that may give rise to hamartomas in the oral cavity. In this case report, we present a case of osseous choristoma arising from the mandibular, posterior buccal gingiva in a young female patient.

## 1. Background

Choristoma is a mass of histologically normal tissue, but it is heterotopic. That said, it exists in an abnormal location. By definition therefore, choristoma is a benign lesion [1]. Based on the tissues they constitute, oral choristomas can be classified as follows: salivary gland choristoma (central or gingival), cartilaginous choristomas, osseous choristomas, lingual thyroid choristoma, lingual sebaceous choristoma, glial choristoma, and gastric/respiratory mucosal choristoma (solid or cystic) [2]. In its turn, osseous choristoma is a tumor-like growth of the lamellar bone [3], covered by a dense fibrous connective tissue lined by the stratified squamous epithelium [3–5], affecting the tongue most [4–6]. With regard to the pathogenesis of osseous choristomas, they are either developmental (arising from ectopic mesenchymal cells from embryonic branchial arches) or posttraumatic growth with subsequent calcification. The posttraumatic mechanism seems true regarding osseous choristomas of the buccal mucosa and the anterior tongue, while the developmental mechanism might be ascribed the remnants of the branchial arch. Otherwise, the anatomical location of the thyroid gland in the development stage might explain the occurrence of such lesions in the posterior third of the dorsum of the tongue, close to the foramen caecum and circumvallate papillae [2]. Krolls et al. were the first who described oral osseous choristomas [3]. Females have higher predilection by 4 times to be affected with osseous choristma, with most cases occurring at younger than 40 years old [7, 8]. It seems that the tongue is the preferred site for development of an intraoral choristoma, with most cases affecting the posterior half of the dorsal surface of the tongue [7–9]. In one review study, the oral and para-oral distributions of osseous choristomas were as follows: tongue (76–78%), buccal mucosa (14–15%), alveolar mucosa (2%), submandibular region (2%), submental region (1%), masseter muscle (1%), and hard plate (1%) [7, 9]. Arising of the osseous choristoma from the periodontium, gingiva, or alveolar mucosa has been scarcely reported. In fact, there has been only a single report of a case with osseous choristoma which originated from the gingiva [10]. Up to our knowledge, this case report will be the second; it presents a case of a female patient presented with osseous choristoma which originated from the gingiva.

## 2. Case Report

An 11-year-old female patient, who was otherwise healthy, was reported to the oral surgery clinic at Leabaib Health Center (PHCC, Qatar) complaining of an overgrowth which developed 1 year ago in the lower left side of the mandible opposite to the first permanent molar region. The mass was asymptomatic but concerning. The patient reported she noticed the growth 1 year ago, and during that period, the lesion increased in size very slowly. The patient could not remember the history of trauma and did not report difficulty in mastication and speech.

Extraoral examinations neither revealed facial asymmetry, extraoral swelling, nor palpable lymph nodes in the submandibular and submental regions.

Upon intraoral examination, there was a nodular lesion extending over the buccal gingival of the lower left first molar mesiodistally. The said tooth was clinically sound. The lesion was pink in color with some erythematous surface, not ulcerated, well-circumscribed, pedunculated, firm to hard on palpation, but not tender, and nonpulsatile. The growth seemed free from the periosteum (not attached to it).

**Figure 1:**
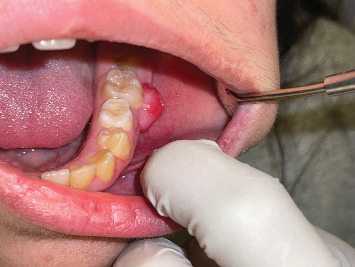
Firm to hard pedunculated growth, pink in color with erythema, originated from the buccal gingiva of the first permanent molar region in the left mandible.

Figure 1 depicts the lesion at time of the first presentation.

**Figure 2:**
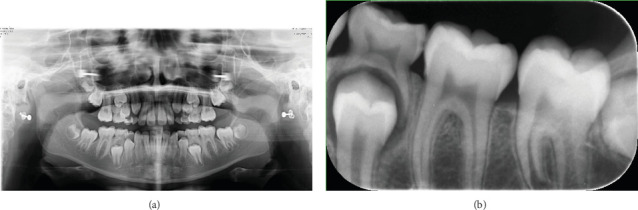
(a) The radiographic findings in the panorama image were insignificant. (b) The radiographic findings in periapical image were insignificant.

The radiographic findings in the panorama and periapical images were insignificant (Figure 2a,b).

The clinical provisional diagnosis was pyogenic granuloma. The differential diagnosis included peripheral ossifying fibroma, peripheral giant cell granuloma, and irritational fibroma.

**Figure 3:**
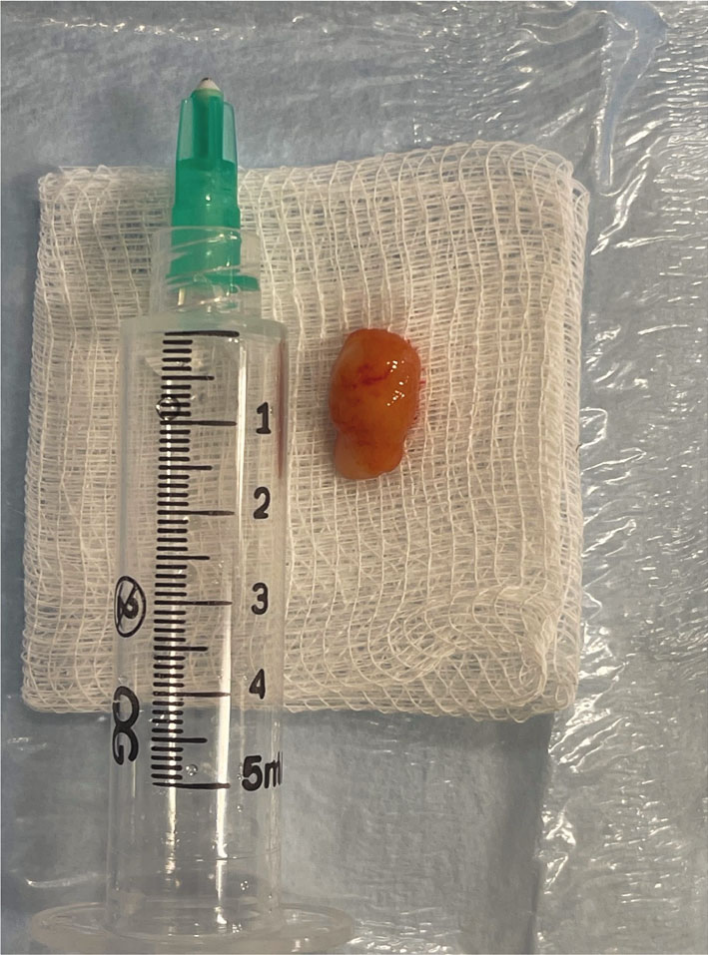
The excised specimen.

After a written consent was obtained from the patient's guardian, the treatment decision was to excise the lesion surgically. The procedure was done under local anesthesia. The approximate dimensions of the excised lesion were 1.3 cm × 0.8 cm × 0.5 cm. The excised specimen consisted of tan nodular and firm tissue (Figure 3). This excisional biopsy was stored in 10% formalin and sent for histopathological examination.

**Figure 4:**
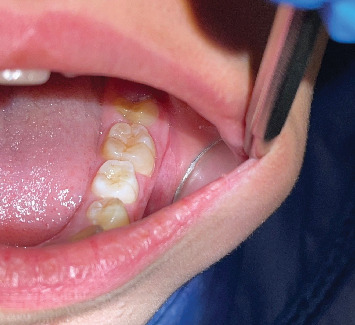
Postoperative at 6 months.

The patient attended for 2 weeks, 1 month, 3 months, and 6 months after treatment. The healing was optimal, and no residual lesion was noted (Figure 4).

### 2.1. Pathological Description

**Figure 5:**
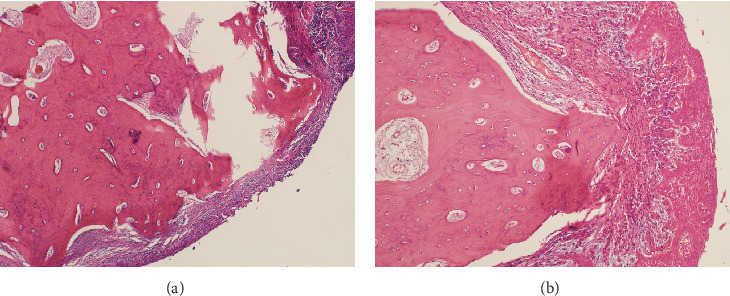
(a) Light microscopic examination revealed a bony lesion in the center covered by epithelium at the periphery. The lesion consists of an anastomosing trabeculae of the cancellous bone formed by osteocytes within the lacunae that are embedded within the osteoid matrix (H&E ×20). (b) Photomicrograph showing the details of the choristoma which consists of plenty of osteocytes embedded in the osteoid matrix. Notice the stratified squamous epithelium at the periphery of the lesion (H&E ×100).

Microscopic examination revealed a well-circumscribed bony lesion covered by oral stratified squamous mucosa (Figure 5). The lesion consists of an anastomosing trabeculae of the cancellous bone formed by osteocytes within the lacunae that are embedded within the osteoid matrix. There were plenty of osteocytes embedded in the osteoid matrix. The lesion was covered by stratified squamous epithelium (Figure 5).

## 3. Discussion

This is the second case, up to our knowledge, of an osseous choristoma originating from the gingiva. The first case was reported by Goswamy et al. [10]. However, our case was the first to report the development of an osseous choristoma from the buccal gingiva, as the case of Goswamy et al. originated from the lingual mucosa.

The intraoral osseous choristomas are uncommon. Although the occurrence of osseous choristomas in the gingiva seems extremely rare, all types of choristoma must be considered among the differential diagnosis of oral masses. The choristoma of the salivary gland type seems more common in the gingiva [11–13]. In our case, we had doubts about peripheral ossifying fibroma, peripheral giant cell granuloma, irritational fibroma, and fibrous hyperplasia because these lesions have anatomical predilection to the gingiva. Given that the surface of the lesion in our case was neither papillary nor verrucous, despite the lesion being pedunculated, neither papilloma, nor verruca vulgaris was included in the differential diagnoses.

Although most patients are unaware of the lesion, many different signs and symptoms like pain, dysphagia, choking gagging, and nausea have been reported in the literature, especially when the tongue is the affected site [14]. In this presented case, the lesion was asymptomatic, and the main concern was the terrifying slow enlargement of the lesion. Clinically, osseous choristoma develops as a firm pedunculated nodular, mostly painless, lesion between 0.5 and 2 cm in size.

The current case was aged 11 years. As most choristomas are developmental, it is logical to find that many of the reported cases affect young ages [2, 15]. Further, our patient did not remember any history of trauma, and the clinical examination precluded any intraoral source of trauma. Both together confirm that the lesion is developmental. However, the presence of an unremembered and/or unidentified source of trauma cannot be totally excluded. A recent review reported a mean age for the reported cases as 31.97 ± 18.04, ranging from 1 month to 89 years old [16], with the majority being between the ages of 20 and 40 years [14].

Moreover, our patient was female. This is consistent with the reported statistics that most choristomas have predilection to female, approaching 2.6:1 according to a recent publication [16].

Osseous choristomas can be easily misdiagnosed as osteomas. However, these two lesions can be differentiated based on their location, their recurrence after excision, and their histological profiles. Basically, osteoma is a benign neoplasm affecting the bone that is associated with normal skeletal structures. Contrastingly, osseous choristoma is a normal bone but found in an abnormal site away from any normal osseous tissue [17–19].

What further confirms the above debate is the histopathological finding in our case. Histologically, choristoma consists of an anastomosing trabeculae of the cancellous bone formed by plenty of osteocytes within the lacunae that are embedded within the osteoid matrix, and the periphery of the lesion is covered by stratified squamous epithelial cells. The latter description for a lesion in a nonbony site is typical of osseous choristoma.

The intraoral choristoma is treated by means of surgical excision [5, 14]. All the cases reported in the literature (139 reported cases) were treated accordingly with only three of them recurring; two of them were on the buccal mucosa and one in the masseter muscle [16]. Such a recurrence scenario may support the notion of traumatic etiology in the development of such lesions. That said, the traumatic causes of development are present in a small fraction of these lesions. Therefore, long-term and close follow-up is mandatory.

## 4. Conclusion

This case report presented a rare condition in a very rare location, osseous choristoma in the buccal gingiva of the posterior mandible, in an 11-year-old female patient. Accordingly, including this pathologic entity in the differential diagnosis of gingival overgrowths (and all oral masses) must be mandatory. Further, follow-up is recommended to confirm nonrecurrence.
